# The genome sequence of the common toad, *Bufo bufo *(Linnaeus, 1758)

**DOI:** 10.12688/wellcomeopenres.17298.1

**Published:** 2021-10-20

**Authors:** Jeffrey W. Streicher, Wellcome Sanger Institute Tree of Life programme, Wellcome Sanger Institute Scientific Operations: DNA Pipelines collective, Tree of Life Core Informatics collective, Darwin Tree of Life Consortium

**Affiliations:** 1 Department of Life Sciences, Natural History Museum, London, UK

**Keywords:** Bufo bufo, common toad, genome sequence, chromosomal

## Abstract

We present a genome assembly from an individual male *Bufo bufo *(the common toad; Chordata; Amphibia; Anura; Bufonidae). The genome sequence is 5.04 gigabases in span. The majority of the assembly (99.1%) is scaffolded into 11 chromosomal pseudomolecules. Gene annotation of this assembly by the NCBI Eukaryotic Genome Annotation Pipeline has identified 21,517 protein coding genes.

## Species taxonomy

Eukaryota; Metazoa; Chordata; Craniata; Vertebrata; Euteleostomi; Amphibia; Batrachia; Anura; Neobatrachia; Hyloidea; Bufonidae; Bufo; *Bufo bufo* Linnaeus 1758 (NCBI:txid8384).

## Introduction

The common toad, *Bufo bufo* (Anura: Bufonidae) is widely distributed throughout Europe. It has a biphasic life cycle that includes aquatic, benthic larvae and terrestrial adults. Bufonids like *B. bufo* are notable amongst anurans in that they (1) lack maxillary teeth, (2) have Bidder’s organs, and (3) have paired paratoid glands that contain alkaloid toxins. *Bufo bufo* has been used extensively in comparative vertebrate research including as a model system in sensory biology ( Ewert, 1974). 

Based on populations from mainland Europe, the nuclear genome size of *B. bufo* was previously estimated to be between 5.82 and 7.75 picograms (= 5.69 and 7.58 gigabases; ( Gregory, 2021)). This is slightly larger than our 5.04 gigabase assembly. The eleven pseudomolecules in our assembly match the expected number of chromosomes in *B. bufo* (2N = 22; six macro- and five micro-chromosomes; ( Birstein & Mazin, 1982; Makino & Others, 1951).

This is the third nuclear genome sequence to be reported from a bufonid anuran ( Edwards *et al*., 2018; Lu *et al*., 2021). The *B. bufo* reference genome reported here has been used to study pseudogenization of the tooth enamel gene amelogenin in bufonids ( Shaheen *et al*., 2021). The genome of a common toad from the UK is particularly timely as a tool for understanding the dynamics of population declines observed over the last two decades ( Carrier & Beebee, 2003; Petrovan & Schmidt, 2016).

## Genome sequence report

**Figure 1.**
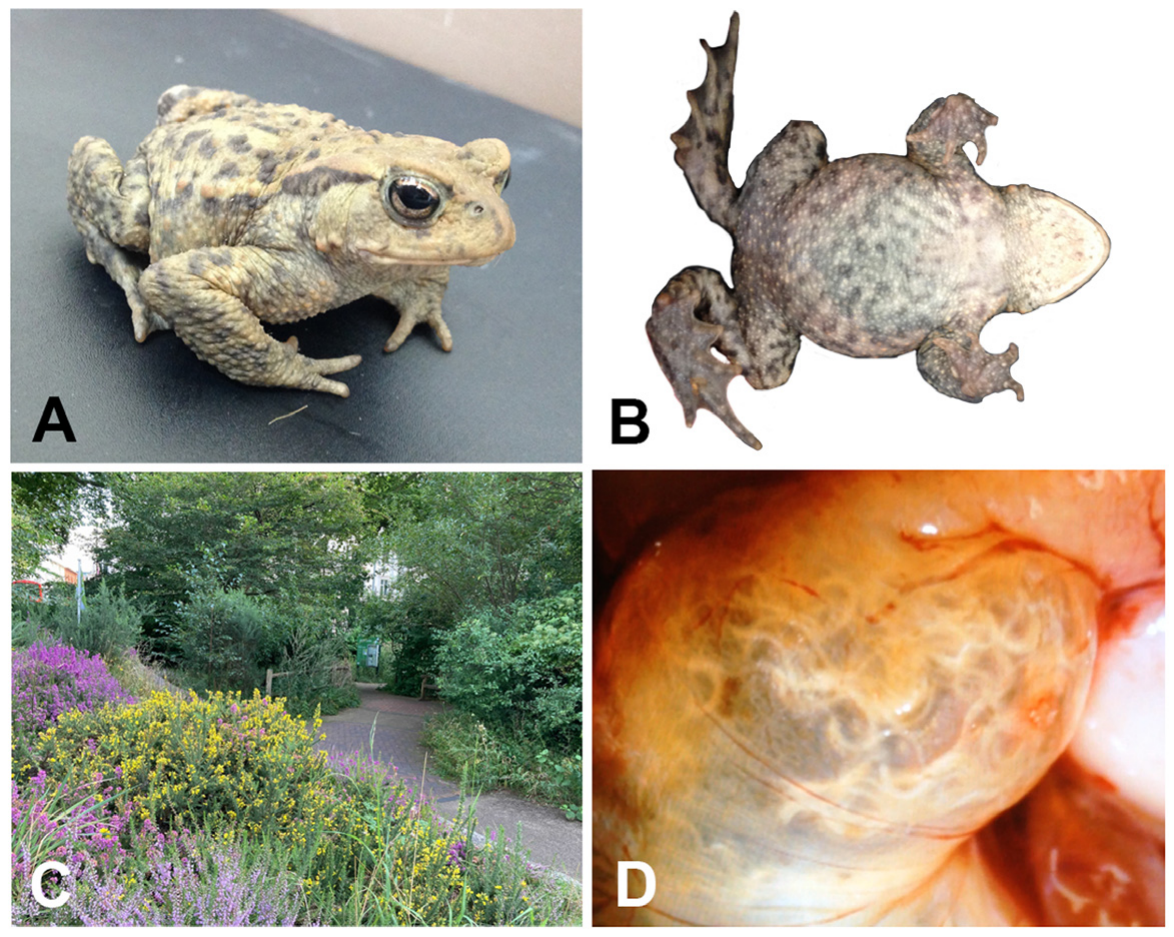
( **A**) Male voucher specimen of *Bufo bufo* (NHMUK 2013.484; Field ID, JWS 758; Snout–Vent Length 55.5 mm) from which the genome was sequenced. ( **B**) Ventral surface of NHMUK 2013.484. ( **C**) The individual was collected from the Natural History Museum Wildlife Garden, London, England. ( **D**) Large numbers of an unidentified nematode parasite were present in the stomach of NHMUK 2013.484.

The genome was sequenced from one male *B. bufo* collected from the Natural History Museum (NHM) Wildlife Garden, London, UK ( Figure 1A, B). A total of 64-fold coverage in Pacific Biosciences single-molecule long reads (N50 28 kb) and 56-fold coverage in 10X Genomics read clouds (from molecules with an estimated N50 of 29 kb) were generated. Primary assembly contigs were scaffolded with chromosome conformation Hi-C data. Manual assembly curation corrected 3498 missing/misjoins and removed 513 haplotypic duplications, reducing the assembly length by 2.4% and the scaffold number by 49.5%, and increasing the scaffold N50 by 38.9%.

**Figure 2.**
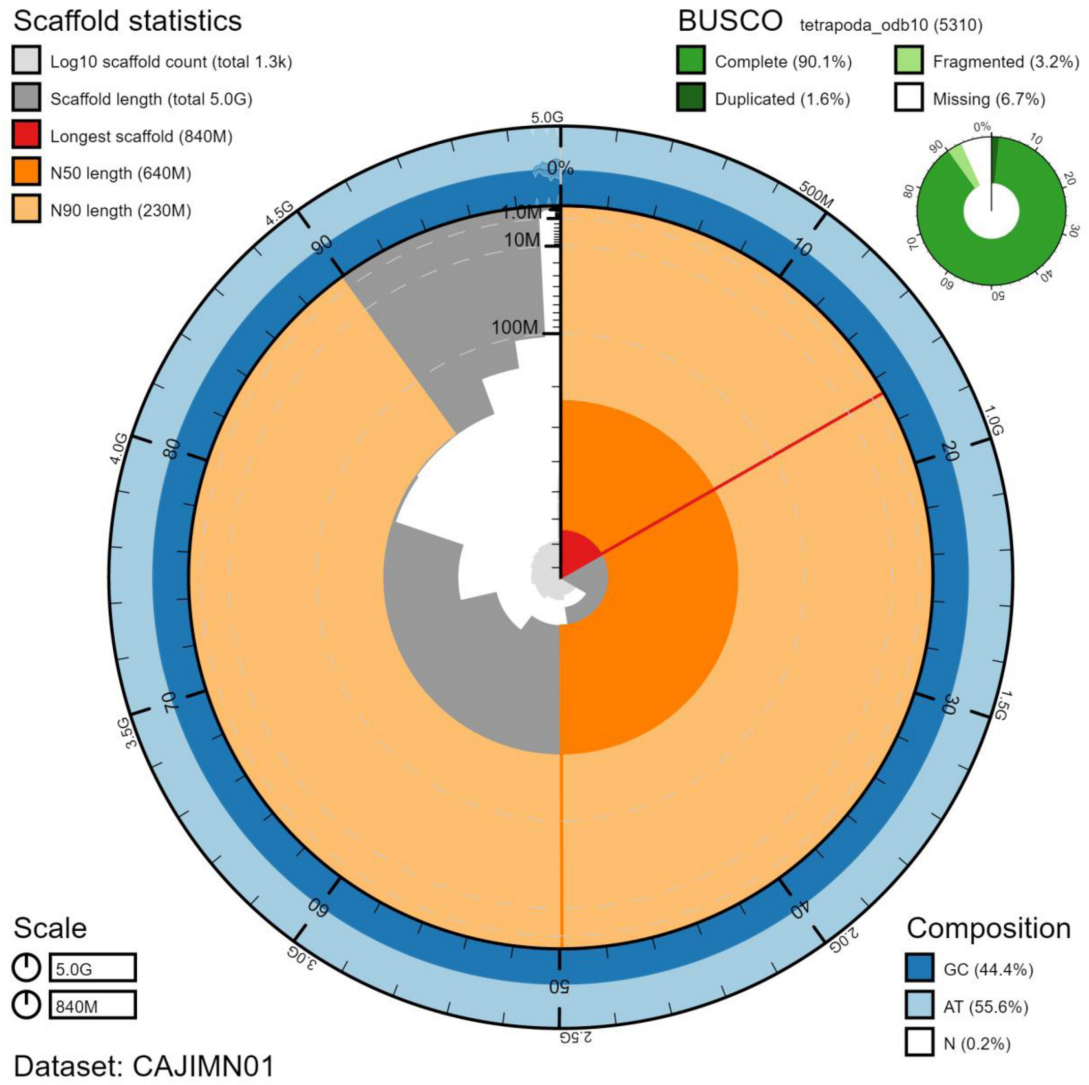
Genome assembly of *Bufo bufo*, aBufBuf1.1: metrics. The BlobToolKit Snailplot shows N50 metrics and BUSCO gene completeness. The main plot is divided into 1,000 size-ordered bins around the circumference with each bin representing 0.1% of the 5,044,762,059 bp assembly. The distribution of chromosome lengths is shown in dark grey with the plot radius scaled to the longest chromosome present in the assembly (843,366,180 bp, shown in red). Orange and pale-orange arcs show the N50 and N90 chromosome lengths (635,713,434 and 230,778,867 bp), respectively. The pale grey spiral shows the cumulative chromosome count on a log scale with white scale lines showing successive orders of magnitude. The blue and pale-blue area around the outside of the plot shows the distribution of GC, AT and N percentages in the same bins as the inner plot. A summary of complete, fragmented, duplicated and missing BUSCO genes in the tetrapoda_odb10 set is shown in the top right. An interactive version of this figure is available at https://blobtoolkit.genomehubs.org/view/Bufo%20bufo/dataset/CAJIMN01/snail.

The final assembly has a total length of 5.04 Gb in 1307 sequence scaffolds with a scaffold N50 of 636 Mb ( Table 1). The majority, 99.1%, of the assembly sequence was assigned to 11 chromosomal-level scaffolds (numbered by sequence length) ( Figure 2– Figure 5; Table 2). The assembly has a BUSCO ( Simão *et al*., 2015) v5.1.2 completeness of 90.1% using the tetrapoda_odb10 reference set. However, a BUSCO (v4.0.2) score of 95.3% using the same reference set was obtained for the annotated gene set of the aBufBuf1.1 assembly (see section *Genome annotation*), indicating that the assembly has a high level of completeness and that some genes were missed during BUSCO analysis of the whole genome assembly. While not fully phased, the assembly deposited is of one haplotype. Contigs corresponding to the second haplotype have also been deposited.

**Table 1.** Genome data for *Bufo bufo*, aBufBuf1.1.

*Project accession data*	
Assembly identifier	aBufBuf1.1
Species	*Bufo bufo*
Specimen	aBufBuf1
NCBI taxonomy ID	NCBI:txid8384
BioProject	PRJEB42238
BioSample ID	SAMEA7521636
Isolate information	Male, heart tissue; NHMUK 2013.484
*Raw data accessions*	
PacificBiosciences SEQUEL II	ERR7012639, ERR7015063-ERR7015065
10X Genomics Illumina	ERR6002753-ERR6002766, ERR6003048, ERR6003049
Hi-C Illumina	ERR6002767-ERR6002770
BioNano	ERZ3003198
*Genome assembly*	
Assembly accession	GCA_905171765.1
*Accession of alternate haplotype*	GCA_905171715.1
Span (Mb)	5,045
Number of contigs	5,502
Contig N50 length (Mb)	3.96
Number of scaffolds	1,307
Scaffold N50 length (Mb)	636
Longest scaffold (Mb)	843
BUSCO genome score *	C:90.1%[S:88.5%,D:1.6%],F:3.2%,M:6.7%,n:5310
*Genome annotation*	
Number of genes	30,286
Number of protein-coding genes	21,517
Average length of gene (bp)	57,667
Average number of exons per gene	12
Average exon size (bp)	241
Average intron size (bp)	8,995
BUSCO annotation score **	C:95.3%[S:93.2%,D:2.1%],F:0.7%,M:4.0%,n:5310

C= complete [S= single copy, D=duplicated], F=fragmented, M=missing, n=number of orthologues in comparison. *BUSCO scores based on the terapoda_odb10 BUSCO set using v5.1.2, run on the aBufBuf1.1 genome assembly using BlobToolKit. A full set of BUSCO scores is available at https://blobtoolkit.genomehubs.org/view/aBufBuf1.1/dataset/CAJIMN01/busco. **BUSCO scores based on the terapoda_odb10 BUSCO set using v4.0.2, run on the NCBI RefSeq annotation of the aBufBuf1.1 genome assembly ( NCBI *Bufo bufo* Annotation Release 100).

**Figure 3.**
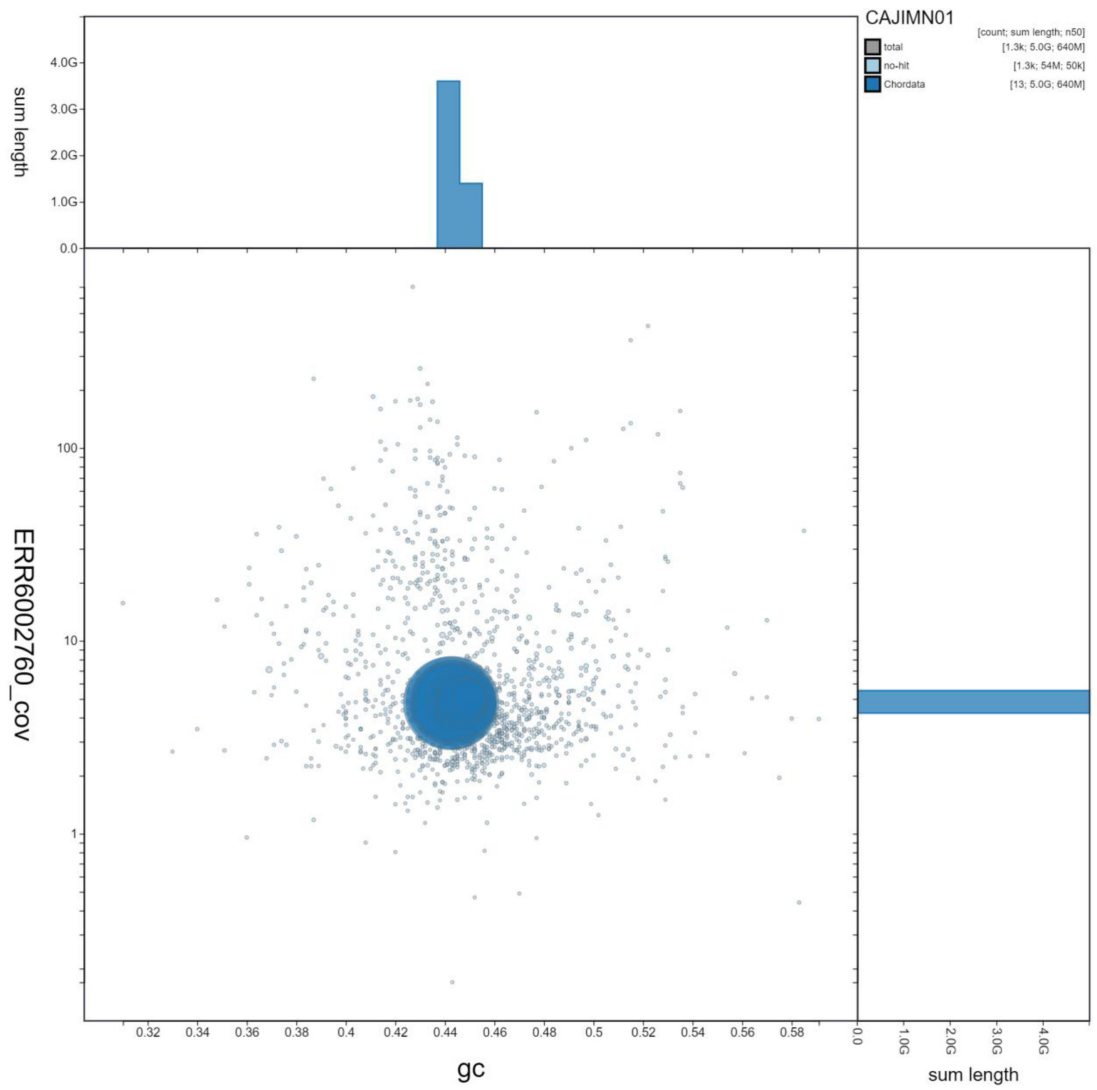
Genome assembly of *Bufo bufo*, aBufBuf1.1: GC-coverage. BlobToolKit GC-coverage plot. Scaffolds are coloured by phylum. Circles are sized in proportion to scaffold length. Histograms show the distribution of scaffold length sum along each axis. An interactive version of this figure is available at https://blobtoolkit.genomehubs.org/view/Bufo%20bufo/dataset/CAJIMN01/blob.

**Figure 4.**
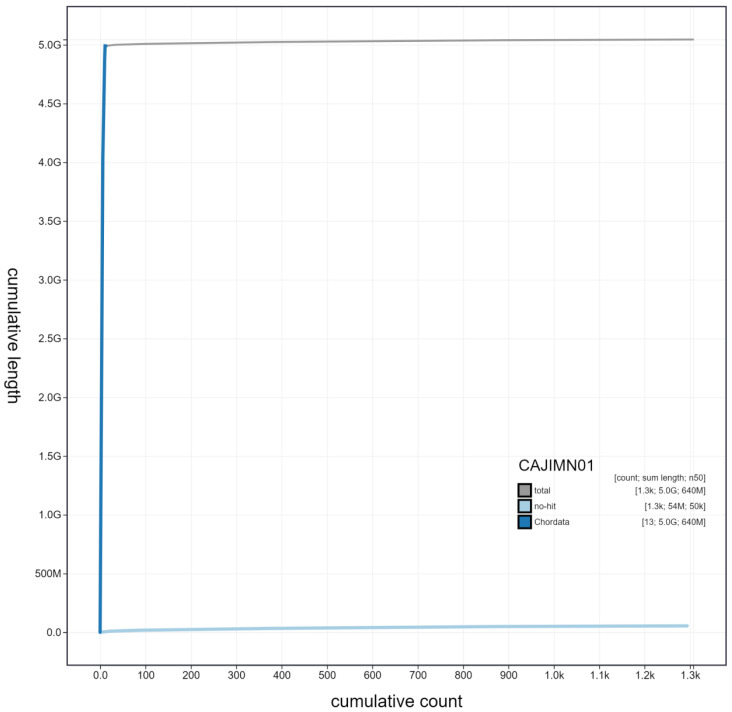
Genome assembly of *Bufo bufo*, aBufBuf1.1: cumulative sequence. BlobToolKit cumulative sequence plot. The grey line shows cumulative length for all scaffolds. Coloured lines show cumulative lengths of scaffolds assigned to each phylum using the buscogenes taxrule. An interactive version of this figure is available at https://blobtoolkit.genomehubs.org/view/Bufo%20bufo/dataset/CAJIMN01/cumulative.

**Figure 5.**
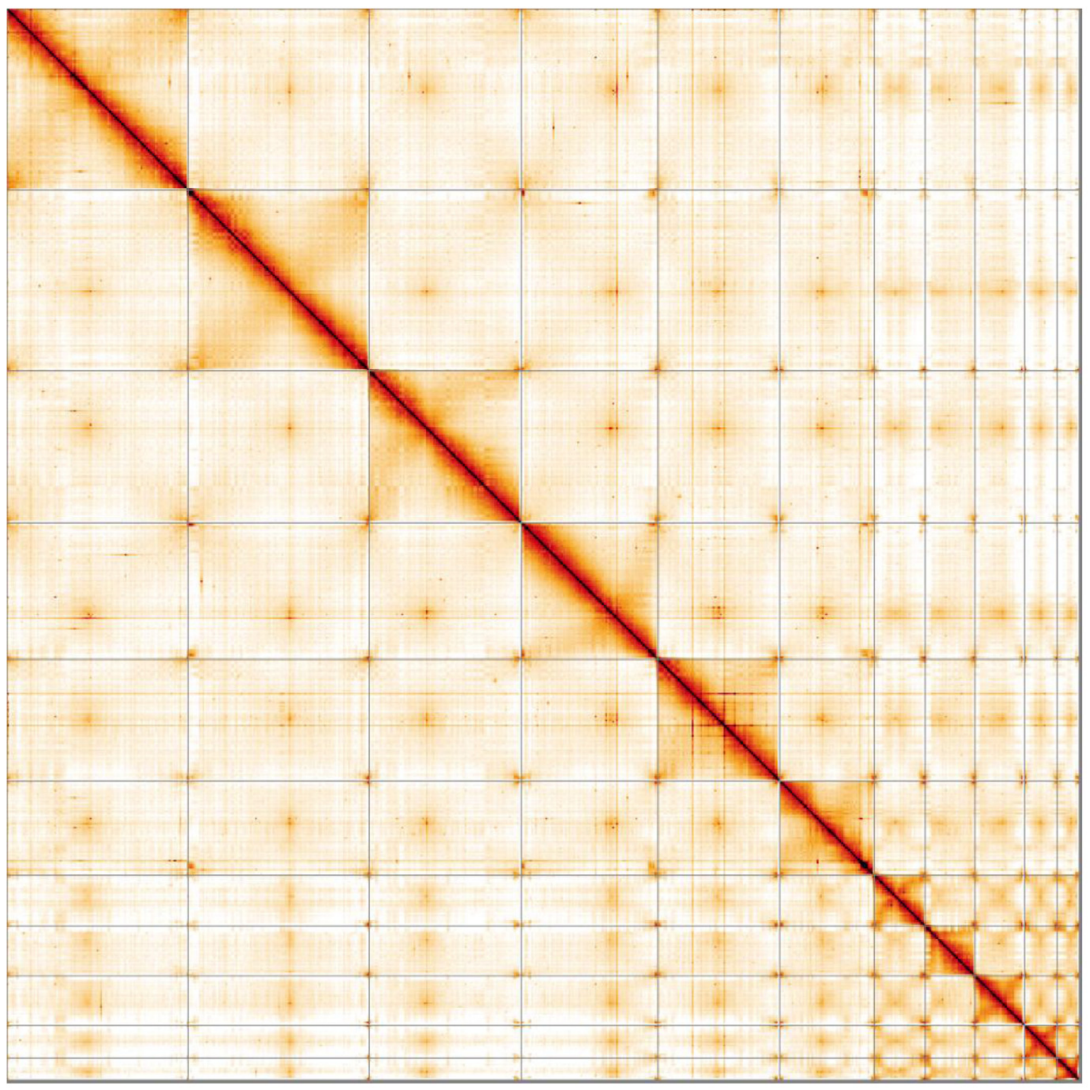
Genome assembly of *Bufo bufo*, aBufBuf1.1: Hi-C contact map. Hi-C contact map of the aBufBuf1.1 assembly, visualised in HiGlass. Chromosomes are presented in size order from left to right and top to bottom.

**Table 2.** Chromosomal pseudomolecules in the genome assembly of *Bufo bufo*, aBufBuf1.1.

INSDC accession	Chromosome	Size (Mb)	GC%
LR991667.1	1	843.37	44.5
LR991668.1	2	842.56	44.3
LR991669.1	3	707.96	44.4
LR991670.1	4	635.71	44.4
LR991671.1	5	567.30	44.4
LR991672.1	6	439.63	44.8
LR991673.1	7	236.60	44.7
LR991674.1	8	231.67	44.8
LR991675.1	9	230.78	44.8
LR991676.1	10	151.57	44.8
LR991677.1	11	103.21	45
LR991678.1	MT	0.02	42.7
-	Unlocalised	54.40	45.1

## Genome annotation

The *B. bufo* assembly was annotated by the NCBI Eukaryotic Genome Annotation Pipeline, an automated pipeline that annotates genes, transcripts and proteins on draft and finished genome assemblies. The annotation ( NCBI Bufo bufo Annotation Release 100; Table 1) was generated from transcripts and proteins retrieved from NCBI Entrez by alignment to the genome assembly, as described ( Pruitt *et al*., 2014).

## Methods

### Sample acquisition

A single male *B. bufo* was collected from a stable, isolated population in the NHM Wildlife Garden, London, UK (latitude 51.49586, longitude -0.178622, elevation 17 m) by Jeffrey W. Streicher on 1 July 2015 ( Figure 1C). The specimen of *B. bufo* (NHMUK 2013.484, Field ID: JWS 758) was 55.5 mm snout–vent length (determined using Miyamoto digital callipers to the nearest 0.1 mm) and contained many nematode parasites in its stomach ( Figure 1D). The specimen was collected with permission from the NHM Wildlife Garden management team and is part of a long-term monitoring project run by the Department of Life Sciences and the Angela Marmont Centre for UK Biodiversity. It was humanely euthanised using a saturated solution of tricaine mesylate (MS-222). Multiple tissues including heart, thigh muscle, liver, eyes, kidney, testes, Bidder’s organ, and intestines were sampled into an ammonium sulfate-based RNA + DNA preservation buffer. After ~24 hours of storage at 4°C, the tissues were transferred to -80°C until they were sent for genome sequencing. Sample tissue has been accessioned by the NHM Molecular Collections Facility (NHMUK 2013.484).

### DNA extraction and sequencing

DNA was extracted from heart tissue using the Bionano Prep Animal Tissue DNA Isolation kit according to the manufacturer's instructions. Pacific Biosciences CLR long read and 10X Genomics read cloud sequencing libraries were constructed according to the manufacturers’ instructions. Hi-C data were generated from heart tissue using the Arima v2 Hi-C kit. Extraction and sequencing was performed by the Scientific Operations DNA Pipelines at the Wellcome Sanger Institute on Pacific Biosciences SEQUEL I and Illumina HiSeq X instruments. DNA was labeled for Bionano Genomics optical mapping following the Bionano Prep Direct Label and Stain (DLS) Protocol and run on one Saphyr instrument chip flowcell.

### Genome assembly

Assembly was carried out following the Vertebrate Genome Project pipeline v1.6 (( Rhie *et al*., 2021)) with Falcon-unzip ( Chin *et al*., 2016), haplotypic duplication was identified and removed with purge_dups ( Guan *et al*., 2020) and a first round of scaffolding carried out with 10X Genomics read clouds using scaff10x. Hybrid scaffolding was performed using the BioNano DLE-1 data and BioNano Solve. Scaffolding with Hi-C data (( Rao *et al*., 2014)) was carried out with HiLine, then 3D-DNA ( Dudchenko *et al*., 2017). The Hi-C scaffolded assembly was polished with arrow using the PacBio data, then polished with the 10X Genomics Illumina data by aligning to the assembly with longranger align, calling variants with freebayes ( Garrison & Marth, 2012) and applying homozygous non-reference edits using bcftools consensus. Two rounds of the Illumina polishing were applied. The mitochondrial genome was assembled at The Rockefeller University using the mitoVGP pipeline ( Formenti *et al*., 2021). The assembly was checked for contamination and corrected using the gEVAL system ( Chow *et al*., 2016; ( Howe *et al*., 2021)). Manual curation was performed using evidence from Bionano (using the Bionano Access viewer), using HiGlass ( Kerpedjiev *et al*., 2018) and Pretext, as described previously ( Howe *et al*., 2021). Figure 2– Figure 4 and BUSCO values were generated using BlobToolKit ( Challis *et al*., 2020). Table 3 contains a list of software tools and versions, where applicable.

**Table 3.** Software tools used.

Software tool	Version	Source
Falcon-unzip	falcon-kit 1.4.2	( Chin *et al*., 2016)
purge_dups	1.0.0	( Guan *et al*., 2020)
scaff10x	4.2	https://github.com/wtsi-hpag/Scaff10X
Bionano Solve	3.3_10252018	https://bionanogenomics.com/downloads/bionano-solve/
3D-DNA	180922	( Dudchenko *et al*., 2017)
Arrow	gcpp 1.9.0-SL-release- 8.0.0+1-37-gd7b188d	https://github.com/PacificBiosciences/GenomicConsensus
Longranger	2.2.2	https://support.10xgenomics.com/genome-exome/software/pipelines/latest/advanced/other-pipelines
freebayes	v1.3.1-17-gaa2ace8	( Garrison & Marth, 2012)
bcftools- consensus	1.10.2	http://samtools.github.io/bcftools/bcftools.html
gEVAL	N/A	( Chow *et al*., 2016)
HiGlass	1.11.6	( Kerpedjiev *et al*., 2018)
PretextView	0.1	https://github.com/wtsi-hpag/PretextView
BlobToolKit	2.6.1	( Challis *et al*., 2020)

### Ethical/compliance issues

The materials that have contributed to this genome note were supplied by a Tree of Life collaborator. The Wellcome Sanger Institute employs a process whereby due diligence is carried out proportionate to the nature of the materials themselves, and the circumstances under which they have been/are to be collected and provided for use. The purpose of this is to address and mitigate any potential legal and/or ethical implications of receipt and use of the materials as part of the research project, and to ensure that in doing so we align with best practice wherever possible.

The overarching areas of consideration are:

- Ethical review of provenance and sourcing of the material;
- Legality of collection, transfer and use (national and international).

Each transfer of samples is undertaken according to a Research Collaboration Agreement or Material Transfer Agreement entered into by the Tree of Life collaborator, Genome Research Limited (operating as the Wellcome Sanger Institute) and in some circumstances other Tree of Life collaborators.

## Data availability

European Nucleotide Archive: Bufo bufo (common toad). Accession number PRJEB42238; https://identifiers.org/ena.embl/PRJEB42238.

The genome sequence is released openly for reuse. The *B. bufo* genome sequencing initiative is part of the Darwin Tree of Life (DToL) project and the Vertebrate Genomes Project. All raw sequence data and the assembly have been deposited in INSDC databases. Raw data and assembly accession identifiers are reported in Table 1.

## Author information

Members of the Wellcome Sanger Institute Tree of Life programme collective are listed here: https://doi.org/10.5281/zenodo.5377053.

Members of Wellcome Sanger Institute Scientific Operations: DNA Pipelines collective are listed here: https://doi.org/10.5281/zenodo.4790456.

Members of the Tree of Life Core Informatics collective are listed here: https://doi.org10.5281/zenodo.5013542.

Members of the Darwin Tree of Life Consortium are listed here: https://doi.org/10.5281/zenodo.4783559.
