# Supplementary material for: The genome sequence of the common toad, Bufo bufo (Linnaeus, 1758)
Source: Wellcome Open Res. 2021 Oct 20;6:281. [Version 1] doi: 10.12688/wellcomeopenres.17298.1 (PMC8729185; doi:10.12688/wellcomeopenres.17298.1)
Supplement: Peer review history

# Peer review: Reviewer response for version 1

**DOI:** 10.21956/wellcomeopenres.19125.r46560

**Published:** 2022-01-04

**Authors:** H. Bradley Shaffer

**Affiliations:** 1 Department of Ecology and Evolutionary Biology, UCLA La Kretz Center for California Conservation Science, Institute of the Environment and Sustainability, University of California, Los Angeles, Los Angeles, CA, USA

This is a straightforward genome release report of an important European anuran. The methods and presentation of the data are obviously formulaic, but in this case, that’s fine. It could be indexed as is, although I have a few small comments that might increase its visibility or clarity, particularly for the amphibian community that will be its primary user group.

 The Introduction is very thin and not really very informative or compelling. I would include more on the family Bufonidae, for example. It is the third most speciose family out of 54 currently recognized on Amphibiaweb, there’s been a lot of recent controversy over genus-level taxonomy - that kind of thing. I’d like to see a bit more on conservation, given how hard toads have been hit by chytrid. What about the portion of the family phylogeny that is now covered by the three species with genomes, and important targets for additional lineages?

**Methods:**

- Usually one records snout-urostyle length in an anuran, rather than snout-vent. Or at least that’s the measurement I prefer. They are almost identical.
- I could live without Figure 4, but it’s fine if that is what you want. I don’t think it’s terribly necessary.

**Small notes:**

- Benthic larvae - I’ve never heard that term for anuran larva. Unless they are very specialized, it applies to pretty much all tadpoles. I would drop this.
- Last sentence of the Genome Annotation section should be corrected to say “…as described by Pruitt *et al. *(2014):
*"The annotation (NCBI Bufo bufo Annotation Release 100; Table 1) was generated from transcripts and proteins retrieved from NCBI Entrez by alignment to the genome assembly, as described by (Pruitt et al., 2014)."*

Are sufficient details of methods and materials provided to allow replication by others?

Yes

Is the rationale for creating the dataset(s) clearly described?

Partly

Are the datasets clearly presented in a useable and accessible format?

Yes

Are the protocols appropriate and is the work technically sound?

Yes

Reviewer Expertise:

Amphibian and reptile evolution and ecology; conservation genomics and genetics; conservation biology

I confirm that I have read this submission and believe that I have an appropriate level of expertise to confirm that it is of an acceptable scientific standard.

# Peer review: Reviewer response for version 1

**DOI:** 10.21956/wellcomeopenres.19125.r46626

**Published:** 2021-11-01

**Authors:** Zexi Cai

**Affiliations:** 1 Center for Quantitative Genetics and Genomics, Aarhus University, Tjele, Denmark

The authors presented a high quality genome assembly of the common toad. The data generation and analysis pipelines applied in this report are sound and the information for the resource reuse are clearly stated in the report. 

 However, in the "Genome annotation" part, the authors stated the method which should go to method part instead of a summary of the annotated genomic feature. Also, in the method part, the detail of running BUSCO should be stated clearly.

Are sufficient details of methods and materials provided to allow replication by others?

Yes

Is the rationale for creating the dataset(s) clearly described?

Yes

Are the datasets clearly presented in a useable and accessible format?

Yes

Are the protocols appropriate and is the work technically sound?

Yes

Reviewer Expertise:

Bioinformatics, quantitative genetics and genomics.

I confirm that I have read this submission and believe that I have an appropriate level of expertise to confirm that it is of an acceptable scientific standard.
